# Aims, Study Design, and Enrollment Results From the Assessing Predictors of Infant Respiratory Syncytial Virus Effects and Severity Study

**DOI:** 10.2196/12907

**Published:** 2019-06-06

**Authors:** Edward E Walsh, Thomas J Mariani, ChinYi Chu, Alex Grier, Steven R Gill, Xing Qiu, Lu Wang, Jeanne Holden-Wiltse, Anthony Corbett, Juilee Thakar, Lauren Benoodt, Matthew N McCall, David J Topham, Ann R Falsey, Mary T Caserta

**Affiliations:** 1 University of Rochester School of Medicine and Dentistry Rochester, NY United States

**Keywords:** respiratory syncytial virus, innate immunity, T-lymphocytes, immunoglobulins, gene expression, transcriptome, microbiota

## Abstract

**Background:**

The majority of infants hospitalized with primary respiratory syncytial virus (RSV) infection have no obvious risk factors for severe disease.

**Objective:**

The aim of this study (Assessing Predictors of Infant RSV Effects and Severity, AsPIRES) was to identify factors associated with severe disease in full-term healthy infants younger than 10 months with primary RSV infection.

**Methods:**

RSV infected infants were enrolled from 3 cohorts during consecutive winters from August 2012 to April 2016 in Rochester, New York. A birth cohort was prospectively enrolled and followed through their first winter for development of RSV infection. An outpatient supplemental cohort was enrolled in the emergency department or pediatric offices, and a hospital cohort was enrolled on admission with RSV infection. RSV was diagnosed by reverse transcriptase-polymerase chain reaction. Demographic and clinical data were recorded and samples collected for assays: buccal swab (cytomegalovirus polymerase chain reaction, PCR), nasal swab (RSV qualitative PCR, complete viral gene sequence, 16S ribosomal ribonucleic acid [RNA] amplicon microbiota analysis), nasal wash (chemokine and cytokine assays), nasal brush (nasal respiratory epithelial cell gene expression using RNA sequencing [RNAseq]), and 2 to 3 ml of heparinized blood (flow cytometry, RNAseq analysis of purified cluster of differentiation [CD]4+, CD8+, B cells and natural killer cells, and RSV-specific antibody). Cord blood (RSV-specific antibody) was also collected for the birth cohort. Univariate and multivariate logistic regression will be used for analysis of data using a continuous Global Respiratory Severity Score (GRSS) as the outcome variable. Novel statistical methods will be developed for integration of the large complex datasets.

**Results:**

A total of 453 infants were enrolled into the 3 cohorts; 226 in the birth cohort, 60 in the supplemental cohort, and 78 in the hospital cohort. A total of 126 birth cohort infants remained in the study and were evaluated for 150 respiratory illnesses. Of the 60 RSV positive infants in the supplemental cohort, 42 completed the study, whereas all 78 of the RSV positive hospital cohort infants completed the study. A GRSS was calculated for each RSV-infected infant and is being used to analyze each of the complex datasets by correlation with disease severity in univariate and multivariate methods.

**Conclusions:**

The AsPIRES study will provide insights into the complex pathogenesis of RSV infection in healthy full-term infants with primary RSV infection. The analysis will allow assessment of multiple factors potentially influencing the severity of RSV infection including the level of RSV specific antibodies, the innate immune response of nasal epithelial cells, the adaptive response by various lymphocyte subsets, the resident airway microbiota, and viral factors. Results of this study will inform disease interventions such as vaccines and antiviral therapies.

## Introduction

### Background

Respiratory syncytial virus (RSV), a negative strand ribonucleic acid (RNA) virus in the *pneumoviridae* family, is the most important cause of respiratory tract infection during infancy, causing annual winter outbreaks lasting 18 to 24 weeks in the United States [1-5]. In the United States, 50 to 70% of the 4 million newborns each year are infected during their first winter, and 1 to 3% are hospitalized, 4 to 7% are seen in emergency departments, and 10 to 16% require physician office visits because of RSV [6]. Although mortality is low in the United States (approximately 50 deaths annually), in developing countries RSV is estimated to cause as many as 118,000 deaths, 6 million cases of severe acute lower respiratory illness, and 3 million hospitalizations annually in children younger than 5 years [7,8]. In addition, severe RSV infection has been linked to development of asthma and implicated recently in development of chronic obstructive lung disease [9-12].

Major risk factors for severe illness include prematurity, cyanotic congenital heart disease, severe neuromuscular disease, immune compromise, and bronchopulmonary dysplasia [13,14]. However, approximately 70% of hospitalized infants have no overt risk factors for severe illness, although young age at infection, environmental factors (secondhand tobacco smoke, lack of breast feeding, household crowding, and low socioeconomic status), and viral, host genetic, and immune factors have been associated with severe disease [14,15]. In addition, high RSV viral load has also been associated with more severe disease in several but not all studies [16], and although group A RSV strains are more common than group B strains among hospitalized infants, the relationship of viral genetic differences to disease severity has not been demonstrated conclusively [17-22].

RSV is not considered highly cytopathic in airway epithelium, whereas host immune responses are thought to be a major contributor to disease pathogenesis [23,24]. Single nucleotide polymorphisms in a number of cytokine and chemokine genes (IL-1, IL-4, IL-8, IL-13, IL-18, RANTES, CCR5), Toll-like receptor 4, and vitamin D receptor have been associated with increased risk of severe disease [25-28]. In contrast, high levels of cord blood neutralizing antibody are associated with delayed onset of hospitalization with RSV and diminished illness severity [29-31]. High levels of maternally derived RSV-specific antibody at infection have also been associated with diminished illness severity in 2 recent reports [31,32]. The most compelling evidence of the beneficial effect of antibody comes from studies of prophylactic administration of immunoglobulin with high titers of RSV neutralizing antibodies or monoclonal antibody to high-risk infants that demonstrate approximately 50% reductions in hospitalization from RSV [33,34]. Some studies suggest that a type 2 helper (Th2)–biased response during primary infection may also be a contributing factor to disease severity [35-37]. Finally, more severe RSV disease has been associated with greater abundance of *haemophilus influenzae and streptococcus pneumoniae* in the nasal microbiota during RSV infection [38-40].

### Objectives

The Assessing Predictors of Infant RSV Effects and Severity study was designed to simultaneously measure a number of host demographic, environmental, and innate and adaptive immune parameters in conjunction with viral factors and the respiratory microbiota in relation to disease severity in full-term healthy infants younger than 10 months undergoing primary RSV infection. We hypothesize that assessment of the interplay of these factors will provide insight into the pathophysiology of RSV disease in this population.

## Methods

### Study Design and Setting

The study was performed in Rochester, New York, encompassing 3 winter RSV seasons spanning from 2012 to 2016. To capture the full spectrum of RSV severity from very mild outpatients to hospitalized infants, 3 cohorts of infants were recruited. Inclusion and exclusion criteria are shown in Textboxes 1 and 2. The investigational review boards of the University of Rochester, Highland Hospital, and Rochester General Hospital (RGH) approved the study.

**Inclusion criteria for all cohorts.**

**Inclusion criteria for all infants in the 3 cohorts:**

- Gestational age ≥37 weeks
- Parent/guardian can provide informed consent
- Infant will be available for duration of the study for birth cohort. For the supplemental and hospital cohorts, infants unable to participate in the full study because of logistic problems such as long travel times from home may be enrolled as long as they agree to complete requirements for visit 1.
- Born after the previous May 1.

**Additional criteria for hospital cohort only**

- Acute illness documented to be because of respiratory syncytial virus infection on admission to hospital.

**Exclusion criteria for all cohorts.**

**Exclusion criteria for all infants in the 3 cohorts:**

- Any infant eligible to receive respiratory syncytial virus (RSV) prophylaxis with Palvizumab.
- Presence of underlying neuromuscular disorder (ie, Down syndrome, cerebral palsy).
- Immunosuppressive condition (ie, HIV infection in mother) or use of immunosuppressive medications before RSV infection.
- Presence of malignancy (ie, Wilms tumor).
- Inability to contact for the duration of the study.
- Any other condition deemed to place infant at higher risk for severe RSV infection (ie, neonatal intensive care unit transfer at birth, recurrent aspiration).

**Additional criteria for hospital cohort only**

- Infants hospitalized for apnea only.

### Cohort Enrollment

Birth cohort: Infants were enrolled at birth in the late summer through midwinter (approximately August 15 to February 1). Infants were enrolled at 3 hospitals: the University of Rochester Medical Center’s (URMC’s) Strong Memorial Hospital and Highland Hospital, and RGH.

Supplemental cohort: A second cohort, designated the supplemental cohort, was recruited from infants seen with acute respiratory illness not requiring hospitalization at URMC’s Golisano Children’s Hospital and RGH emergency departments or at pediatric clinics at URMC’s Golisano Children’s Hospital and RGH, and the Elmwood Pediatric Practice, a private pediatric office affiliated with URMC.

Hospital cohort: Infants were enrolled on admission to URMC’s Golisano Children’s Hospital and RGH with documented RSV infection.

### Surveillance for Respiratory Syncytial Virus

Illness surveillance and surveillance visits: Infants enrolled in the birth cohort were followed by passive and active surveillance for development of respiratory symptoms throughout their first winter (mid-November until April 15). Parents were asked to call when their infant developed any of the following symptoms: nasal congestion, nasal discharge, cough, wheezing, sustained rapid breathing, or fever. A study nurse evaluated the infant in the research clinic or at a home visit, and a nasal swab was collected for RSV diagnosis. Supplemental cohort infants had nasal swabs collected at the time of evaluation in the emergency department or physician’s office. Nasal swabs were tested for the presence of RSV RNA using an RSV-specific reverse-transcriptase polymerase chain reaction (RT-PCR) assay or by rapid antigen testing (Quidel) and confirmed by RT-PCR, as described [41]. The hospital cohort infants were identified as RSV-infected by the clinical virology laboratory using either of 2 commercial RT-PCR assays (Focus Simplexa RSV/influenza duplex PCR or the Pasteur Merrieux Biofire multiplex PCR assay).

### Study Visits

**Figure 1:**
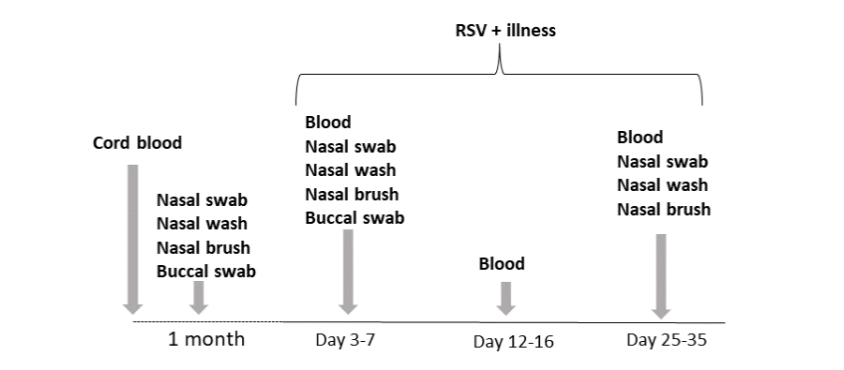
Schematic of chronological procedures for Assessing Predictors of Infant respiratory syncytial virus (RSV) Effects and Severity study. Dotted line represents time course for birth cohort before identification of RSV illness. Solid line represents time course of samples and procedures for RSV positive infants in all 3 cohorts. RSV: respiratory syncytial virus.

The chronology of study visits for the birth cohort (cord blood and 1-month visit) and illness visits for all RSV infected infants in each of the cohorts are exemplified in Figure 1.Enrollment visit**:** At enrollment a study nurse and 1 of the physician investigators (MTC, EEW, ARF) explained study objectives and procedures to the parents and obtained written informed consent. Demographic data were collected including gestational age, birth weight, household size including number and age of siblings or other children, tobacco use by mother/household members, and breastfeeding frequency. For infants in the birth cohort, a cord blood sample and a buccal swab were obtained (Figure 1).

One-month visit: The birth cohort was seen at the age of 1 month at a time when asymptomatic and a nasal swab, nasal wash, nasal brush, and buccal swab was collected.

Acute respiratory Illness visits: When infants in the birth cohort and supplemental cohort were seen for respiratory illness, a nasal swab was obtained for same day RSV diagnosis. If RSV was identified, the infant was scheduled for an RSV positive illness visit within 24 hours at the research clinic or during a second home visit.

RSV positive illness visits: RSV-infected Infants were evaluated at 3 time points: acute visit when RSV was first identified, at 12 to 16 days, and approximately 28 days after illness onset. At the acute illness visit, clinical information was collected (date of illness onset, presence of nasal congestion/discharge, cough, wheezing, rapid breathing, apnea, cyanosis, fever, lethargy, poor feeding). A physical examination was performed noting weight, pulse, respiratory rate, temperature, room air oxygen saturation (SaO2), presence of cyanosis, nasal discharge, rales or rhonchi, wheezes, and chest retractions. Biological samples were collected in the following sequence: buccal swab, nasal swab from 1 nostril, nasal wash followed by nasal brush from the contralateral nostril, and 2 to 3 ml venous blood sample. Parents were given an illness diary card to record signs of illness for a 10-day period beginning retrospectively from illness onset. Medical records of hospitalized infants and those seen in the emergency department or physicians’ offices were reviewed and clinical information recorded. For hospitalized infants the following information was also collected: amount and duration of supplemental oxygen, respiratory support and intensive care requirements, fluid and antibiotic administration, and worst values for each of the respiratory (respiratory rate, pulse, wheezing, rales/rhonchi, retractions, and SaO2) and systemic signs (fever, lethargy, difficulty feeding). Length of hospitalization and results of complete blood count, blood cultures, and chest radiographic findings, if available, were recorded. Interim history and findings on physical examination and room air SaO2 were recorded; a 2 to 3 ml venous blood sample obtained at the second visit; and a nasal swab, nasal wash, nasal brush, and 2 to 3 cc venous blood sample was obtained at the third visit.

### Clinical Study Procedures

Multiple study procedures were performed on enrolled subjects (Table 1).

#### Cord Blood

Cord blood was collected on all birth cohort subjects and the plasma fraction stored at –80^o^C.

#### Buccal Swab

 A buccal swab was collected using a cotton tipped swab at enrollment, the 1-month visit for the birth cohort, and the first RSV-positive illness visit in all cohorts.

#### Diagnostic Nasal Swab

For infants in birth and supplemental cohorts, a viral diagnostic swab was collected at an illness visit using a medium-sized pediatric flocked swab (cat. no. 518CS01, Copan Diagnostics, Murrieta, CA). The swab was placed in 3 ml of UTM viral transport media (Copan Diagnostics) for RT-PCR as described [41].

#### Respiratory Syncytial Virus Illness Visit Nasal Swab

A nasal swab was collected from 1 nostril and placed in 2 ml of ultraviolet-inactivated sterile water. The swab was agitated and removed and the sample split into 2 equal portions and frozen at –80^o^C. The swab sample was used for quantitative RT-PCR and microbiota assays.

**Table 1:** Time and events table for Assessing Predictors of Infant Respiratory Syncytial Virus Effects and Severity study.

Samples/data		Birth cohort visits			RSV^a^ positive illness		
		Birth	1 month	Illness surveillance	Acute (day 2-7)	Day 12-16	Day 25-35
Demographic		X^b^	X	—^c^	X	X	X
Clinical		X	X	—	X	X	X
Cord blood for antibody		X	—	—	—	—	—
Buccal swab for *cytomegalovirus* polymerase chain reaction		X	X	—	X	—	—
Nasal swab RSV RT-PCR^d^		—	—	X	—	—	—
**Nasal swab for:**							
	Quantitative RSV RT-PCR	—	—	—	X	—	X
	Viral coinfection	—	—	—	X	—	X
	RSV gene sequencing	—	—	—	X	—	X
	Microbiome	—	X	—	X	—	X
Nasal wash		—	X	—	X	—	X
Nasal brush for epithelial cell RNAseq^e^		—	X	—	X	—	X
**Blood for:**							
	Peripheral blood mononuclear cells flow cytometry	—	—	—	X	X	X
	CD^f^4, CD8, B, natural killer RNAseq	—	—	—	X	X	—
	Antibody assays	—	—	—	X	X	X

^a^RSV: respiratory syncytial virus.

^b^X indicates samples at this time point.

^c^Indicates no samples collected at time point.

^d^RT-PCR: reverse-transcriptase polymerase chain reaction.

^e^RNAseq: ribonucleic acid sequencing.

^f^CD: cluster of differentiation.

#### Nasal Wash And Nasal Brush

A nasal wash was performed using the opposite nostril as the nasal swab by rapidly instilling and retrieving 5 ml of preservative-free sterile phosphate buffered saline using a small sterile nasal suction bulb, as described [42]. The nasal wash fluid was frozen at –80^o^C. Immediately following the nasal wash, the same nostril was brushed with a medium-sized pediatric flocked swab by rubbing the swab back and forth and rotating it against the middle turbinate mucosa for 5 seconds. The swab was immediately placed in RNA stabilizer (RNAprotect, Qiagen, Germantown, MD) and held at 4°C until cells were recovered by filtering through a 0.45 uM membrane filter. Cells were lysed and homogenized by passing through a 28 g needle, and total RNA was recovered (AbsolutelyRNA Miniprep kit, Agilent, Santa Clara, CA) according to manufacturer’s instructions and stored at –80^o^C.

#### Venipuncture

A total of 2 to 3 mL of blood was collected from an antecubital or hand vein into heparin coated syringes and transferred to a heparinized vacutainer vial. Blood was transported at room temperature to the laboratory within 2 hours for processing.

### Laboratory Procedures, Assays, and Data to Be Generated

RSV RT-PCR: Diagnosis of RSV infection and designation as group A or B RSV was made by RT-PCR using nasal swab samples as previously published [41].

#### Quantitative RSV RT-PCR

Viral load in nasal swab and nasal wash samples was determined using an RSV group–specific quantitative RT-PCR and reported as plaque forming units (pfu)/ml equivalents [43].

#### Respiratory Syncytial Virus Gene Sequences

RNA was extracted from 250 ul of nasal wash or nasal swab specimens as previously described [41]. Full genome sequence of RSV was produced by reverse transcription and PCR amplification of 4 overlapping genome regions in a method similar to Schobel et al and Bose et al [44,45]. The 4 genome amplicons were paired-end sequenced using Nextera XT and Illumina V3 chemistry on a MiSeq (Illumina, San Diego, California). The sequencing reads were assembled into genome contigs using the viral-ngs package (version V1.15.3, Broad Institute Viral Genomics), aligned to a curated set of complete RSV genome isolate sequence from Genbank using MUMmer (SourceForge.net)and annotated using VIGOR (SourceForge.net) [46-48].

#### Non-Respiratory Syncytial Virus Reverse-Transcriptase Polymerase Chain Reaction/Polymerase Chain Reaction/

The presence of other respiratory viruses (parainfluenza viruses 1-3, influenza A and B, coronaviruses, human metapneumovirus, rhino/enteroviruses, adenoviruses, bocavirus) in nasal swab or wash samples was determined using a TaqMan Array Card (Applied Biosystems, Waltham, MA), as described [49].

#### Blood

Whole blood was centrifuged at 300 x g for 10 min at 4^o^C and the plasma removed and stored at –80^o^C. Peripheral blood mononuclear cells (PBMCs) were separated by Ficoll-hypaque gradient and approximately 4 million cells set aside for T and B cell lymphocyte subset sorting (below) and the remainder frozen in liquid nitrogen in 90% fetal calf sera/10% deoxymethysulfoxide (DMSO) and frozen in liquid nitrogen for flow cytometry.

#### Peripheral Blood Mononuclear Cell Sorting

PBMC were flow-sorted into 4 subsets (cluster of differentiation [CD]4^+^, CD8^+^, B cells, and natural killer [NK] cells) using published methods [50]. Cells were immediately lysed in RNA protect and stored at –80^o^C.

Flow cytometry: PBMCs were thawed, rested overnight, and assayed for cytokine and surface markers by flow cytometry following stimulation with cell culture grown RSV, overlapping 18-mer peptide pools representing the RSV fusion, attachment, nucleocapsid and matrix proteins dissolved in DMSO, and controls (DMSO alone, uninfected cell culture supernatant, staphylococcal endotoxin B), as described [51].

#### Ribonucleic Acid Purification

RNA was recovered from nasal brushes stabilized in RNAprotect, fresh sorted PBMC, or thawed and restimulated PBMC using the AbsolutelyRNA Miniprep kit (Agilent, Santa Clara, CA), as previously described [37,52].

#### Plasma Immunoglobulin G Titers to Respiratory Syncytial Virus Proteins

IgG titers to purified RSV F, Ga, Gb proteins were determined in by enzyme immunoassay (EIA) as described [41]. IgG titers to the conserved central CX3C containing region of Ga and Gb proteins were determined by competition EIA with a Fab fragment of a murine mab (L9) specific for the conserved central region of the RSV G proteins [31].

#### Neutralization titers to RSV A and B strains

Serum neutralization titers to RSV group A virus (A2 strain) and B virus (B1 virus) were performed using a modification of previous methods [31]. Plasma was first converted to serum by enzymatic digestion of heparin by hepzyme, followed by inactivation of both the enzyme and complement at 56^o^C for 30 min.

Host transcriptomics: RNA sequencing of nasal brush samples and flow-sorted T and B cells (CD4^+^, CD8^+^, and CD19^+^ cells) was performed as previously described [37,52]. Starting with 1 ng of RNA and using the SMARter Ultra Low amplification kit (Clontech, Mountain View, CA), libraries were constructed using the NexteraXT library kit (Illumina, San Diego, California) and sequenced on the Illumna HiSeq2500 to generate approximately 20 million 100 bp single end reads per sample. Preanalysis data processing was as described [52].

#### Host Gene Expression Validation

Quantitative RT-PCR (qPCR) validation of RNAseq-based gene expression estimates were performed as described [37,52]. Nasal washings were used for quantitative EIA analysis of various cytokines and chemokines.

#### Nasal Microbiota Analysis

Total genomic deoxyribonucleic acid (DNA) was extracted by mechanical lysis and 16S ribosomal RNA was amplified with high-fidelity DNA polymerase and dual indexed primers specific to the V3-V4 hypervariable regions as previously described [52]. Amplicons were pooled and paired-end sequenced (2 X 300 nt) on an Illumina MiSeq. Each sequencing run included: (1) positive controls consisting of standardized bacterial genomic DNAs and (2) negative controls consisting of sterile saline. Sequence processing and initial microbial composition analysis were performed with the Quantitative Insights into Microbial Ecology (QIIME) software package, version 1.9.1 [53]. Operational taxonomic units (OTUs) were picked using the reference-based USEARCH (version 5.2) pipeline in QIIME using the May 2013 release of the GreenGenes 99% OTU database as a closed reference [54-56]. Representative OTU sequences used to make taxonomic assignments for each cluster were selected on the basis of abundance. The RDP Naïve Bayesian Classifier was used for taxonomic classification with the GreenGenes reference database, using a minimum confidence threshold of .85 and otherwise default parameters [57].

### Planned Statistical Analyses

Demographic and clinical data will be assessed by descriptive analysis using means and SE, medians and inter-quartile ranges for continuous variables, and proportions for categorical variables. We will use graphical methods such as histograms, Q-Q plots, and box-plots to visualize the data and identify potential data problems such as outliers, missingness, and skewness. For continuous variables, we will test their normality by Shapiro-Wilk test and Kolmogorov-Smirnov test. If problems are detected, appropriate data preparation steps such as outlier removing, data imputation, and log-transformations will be considered. For those variables that pass the normality test, we will perform 1-way analysis of variance (ANOVA) *F* test followed by Bonferroni *post hoc* testing for pairwise group comparisons. When groups exhibit unequal variances, Welch ANOVA method will be used instead. For non-normal variables, nonparametric Kruskal-Wallis test with Dunn *post hoc* test will be used instead. Extended Fisher exact test will be used to compare the proportions of categorical variables such as gender and race between cohort groups. Pearson and Spearman correlation analysis will be used to assess associations between 2 continuous variables such as severity and gene expression levels. Multivariate linear regression will be used to model the associations between covariates and continuous outcome variables, controlling for possible confounding effects such as age and sex. ANOVA *F* test and regression *t* test will be used to assess the significance of the overall and specific linear association between the covariates and the outcome variables. Multivariate logistic regression will be used to model associations between covariates and categorical outcome variables such as hospitalization, controlling for possible confounding effects. The Wald test will be used to assess significance of the association between covariates of interest and the outcome variables. *P* values <.05 will be considered statistically significant for standard statistical analyses. Some research aims involve analyzing high-throughput data such as RNAseq-based transcriptomic data and 16S-based microbiome data, and we will apply suitable multiple testing procedures, such as the Benjamini-Hochberg procedure, to control false discovery rate at a prespecified level (0.05) [58]. These high-throughput data may have high-level of between-sample variations, in part because of technical issues such as technical noise and batch effects. These undesirable variations can be reduced by a combination of stringent quality assurance analysis and specialized data transformation techniques. Specifically, samples with insufficient quality metrics (low read number, mapping rate or poor sample-wide correlation) will be excluded from analysis. Before statistical analysis, we will explore the dataset by principle component analysis (PCA), principal coordinates analysis, and hierarchical clustering to identify any unwarranted structure or association. If necessary, batch correction methods such as ComBat [59] and surrogate variable analysis [60] may be applied. Due to the non-normal nature of these data, specialized normalization methods and analytical pipelines will be used whenever appropriate [53,60-69]. All statistical analyses will be performed in R 3.3.0 (R Foundation for Statistical Computing, Vienna, Austria) and SAS 9.3 (SAS Institute).

Primary hypothesis: Our primary hypothesis is that we will be able to identify a number of factors associated with disease severity during primary RSV infection in full-term healthy infants. The first step toward this goal was to develop a Global Respiratory Severity Score (GRSS) reflecting the severity of the entire illness as the main outcome variable in most of our analyses [70]. PCA and multivariate logistic regression was used to determine the 9 optimal clinical parameters and their relative weights that comprise the GRSS (available as a Web-based algorithm [71]). We will assess each of the following datasets for their association with disease severity: (1) CD4^+^, CD8^+^ T cell, B cell ,and NK cell gene expression during RSV infection; (2) nasal epithelial cell gene expression before, during, and after RSV infection; (3) CD4^+^ and CD8^+^ cytokine synthesis as measured by flow cytometry during and after RSV infection; (4) serum antibody to RSV F and G proteins and the conserved central CX3C G protein region; (5) serum neutralizing titers to RSV A2 and B1 viruses during and after infection; (6) nasal microbiota composition before, during, and after RSV infection; (7) viral load; and (8) complete RSV genomic sequences. Each analysis will be adjusted for confounding demographic variables such as sex, gestational age, breast feeding history, age at infection, history of exposure to tobacco smoke, coinfection with other respiratory viruses, and the presence of pathogenic bacteria such as *streptococcus pneumoniae, haemophilus influenzae,* and *moraxella catarrhalis* as determined by direct PCR. Most importantly, we will seek to integrate several datasets in complex analyses to assess various interactions between virus, nasal epithelial cell and T cell gene expression, nasal microbiota, and adaptive immune responses as they relate to disease severity. Due to the inclusion of multiple high-dimensional datasets, we plan to assemble novel data integration pipelines that use state-of-the-art dimension reduction methods such as multi-omics factor analysis [72] and multi-set canonical correlation analysis [73] to extract the most informative features from individual high-throughput datasets, and afterwards, use statistical learning techniques such as penalized regression [74-76] and support vector machine classification [77] to predict disease severity. Strict cross-validation criterion will be used to evaluate the performance of the proposed methods. Recently, we developed FUNNEL, which is a time-course gene set enrichment method that has the capability to incorporate both between-gene correlation and weights [78]. We propose to design a new weighting method based on supervised principal component analysis [79] and extend FUNNEL for cross-sectional data and use this new method to study the biological functions of key genes with the largest absolute loadings in the extracted principle components/canonical vectors.

## Results

### Recruitment and Enrollment

During the 3 seasons, 226 infants were enrolled in the birth cohort. Of these, 126 (55.8%) attended the scheduled 1-month visit, indicating a relatively high early attrition rate. Following the 1-month visit, 150 respiratory illnesses were reported in 81 infants (64% of active subjects) during their first winter season, each of which was evaluated during a home visit or clinic visit. A total of 36 infants had a single illness, 29 had 2 illnesses, 11 had 3, and 5 had 4 or more illnesses. A total of 19 of the 81 (23.5%) evaluated infants were shown to be RSV positive, and the overall incidence of RSV infection in the active birth cohort was 15% (19/126). Among the 19, 4 were hospitalized during the RSV infection, 1 of whom was admitted to the pediatric intensive care unit (PICU) for noninvasive respiratory support.

The supplemental cohort was recruited during the second and third winter seasons when it was apparent that the early withdrawal rate in the birth cohort was higher than anticipated. A total of 149 infants with respiratory illness met inclusion/exclusion criteria for the supplemental cohort. Of these, 60 (41%) were documented to have RSV infection, and 42 (70%) were enrolled in the full study. A total of 24 (57%) infants were enrolled from physician offices, whereas 18 (43%) were enrolled from the emergency departments. A total of 2 infants were subsequently hospitalized, 1 of who was admitted to the PICU and intubated.

**Table 2:** Demographic characteristics of 3 cohorts comprising final study populations.

Characteristic		Birth cohort enrolled (n=226)	Birth cohort RSV^a^+ (n=19)	Hospital cohort RSV+ (n=78)	Supplemental cohort RSV+ (n=42)
Male sex, n (%)		119 (53)	9 (47)	36 (46)	20 (48)
**Race, n (%)**					
	White	128 (57)	12 (63)	52 (67)	21 (50)
	Hispanic	39 (17)	3 (16)	9 (12)	8 (19)
Gestational age (weeks), mean (SD)		39.3 (1.1)	38.8 (1.1)	38.8 (1.3)	39.1 (1.4)
Birth weight (kg), mean (SD)		3.3 (0.5)	3.3 (0.7)	3.4 (0.6)	3.3 (0.6)
C-section, n (%)		69 (31)	9 (47)	25 (32)	6 (14)
Mother’s age (years), mean (SD)		28.4 (6.7)	31.3 (5.3)	28.1 (6.0)	29.0 (4.9)
**Household members, mean (SD)**					
	Siblings	1.1 (1.2)	1.4 (1.2)	1.5 (1.2)	1.4 (1.3)
	Adults	2.1 (0.7)	2.0 (0.6)	2.4 (1.2)	2.6 (2.4)
	Other children	0.2 (0.8)	0.1 (0.5)	0.3 (0.7)	0.4 (1.0)
	Total household size	3.4 (1.5)	3.5 (1.3)	4.1 (1.7)	4.1 (1.7)
**Smoking in home, n (%)**					
	Mother	11 (5)	0 (0)	15 (19)	8 (19)
	Others	52 (23)	0 (0)	30 (39)	12 (29)
**Residence, n (%)**					
	House	165 (73)	16 (84)	55 (71)	35 (83)
	Apartment	61 (27)	3 (16)	23 (30)	7 (17)
**Mother’s education**					
	College degree	100 (44)	12 (63)	34 (44)	15 (36)
	High school degree	77 (39)	5 (26)	30 (39)	10 (24)
History of asthma in siblings; no. (%)		29 (21)^b^	2 (14)	20 (32)^b^	14 (47)^b^
Age at RSV infection, mean (SD)		—^c^	2.3 (1.0)	2.9 (2.2)	4.7 (2.0)

^a^RSV: respiratory syncytial virus.

^b^Percentage calculated only for those with siblings in household.

^c^Not applicable.

During 3 seasons, 78 RSV-infected infants meeting inclusion/exclusion criteria were enrolled at the time of hospitalization. A total of 9 were cared for in the PICU and 4 intubated. The average length of stay for all hospitalized infants, including those from the birth and supplemental cohorts, was 4.2 (SD 0.6) days, with a median of 2.3 days. None of the infants died. The demographic characteristics of the RSV-positive subjects in the 3 cohorts are shown in Table 2.

Although for most factors the differences between cohorts were relatively minor (ie, gestational age, mother’s age, household size, number of siblings in the home), there were several significant differences. Birth by cesarean section was significantly lower in the supplemental cohort (6/42) compared with the birth cohort (9/19) and the hospital cohort (25/78; *P*=.001 and *P*=.05, respectively). Maternal smoking was more common in both the supplemental (8/42) and hospital cohorts (15/78) compared with the RSV-infected birth cohort (0/19), both significant (*P*=.05 and *P*=.04, respectively).

A large number of biological samples were collected from the enrolled subjects, including 217 cord blood samples (birth cohort only), 533 buccal swabs, 322 illness blood samples, 661 nasal swabs, 356 nasal wash samples, and 366 nasal brush samples.

### Severity Score Outcome

**Figure 2:**
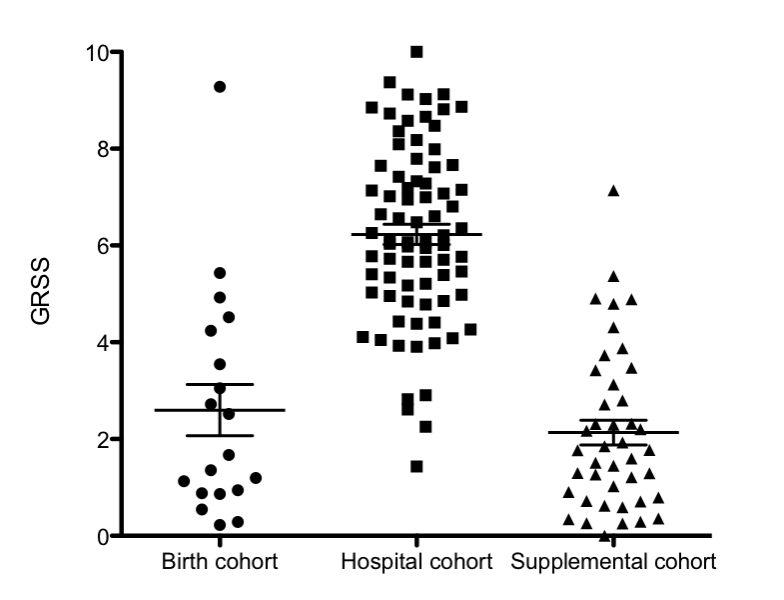
Distribution of the Global Respiratory Severity Score for respiratory syncytial virus–infected infants enrolled in the 3 cohorts. GRSS: Global Respiratory Severity Score.

As noted, we first completed development of the GRSS [70]. The GRSS distribution of the enrolled infants varied by cohort as expected, with lower severity scores in the birth and supplemental cohorts and higher severity score in the hospital cohort (Figure 2).

The mean (SE) GRSS for the birth and supplemental cohorts (both including hospitalized infants from these cohorts) were 2.6 (0.5) and 2.1 (0.3), respectively, and was 6.2 (0.2) for the hospital cohort.

## Discussion

Although conditions such as extreme prematurity, cyanotic heart disease, and immunosuppression have been clearly identified as risk factors for severe RSV infection, the majority of hospitalized infants are normal full-term infants. The pathogenesis of RSV in this population is not completely understood, although a number of additional factors have been suggested as potentially relevant, such as a Th2 bias following birth, genetic variations in regulation of cytokine and chemokine genes, levels of RSV-specific antibodies, variations in nasal microbiota, and viral factors. Our study was designed to simultaneously measure and analyze many of these factors as they relate to severe RSV disease in full-term infants during primary infection. In addition to more severely ill infants hospitalized with RSV infection, we sought to enroll RSV-infected infants spanning the entire spectrum of disease severity by prospectively following a large birth cohort with the expectation that the majority of infections would represent very mild illness.

### Limitations

A comprehensive study of this type, one that requires a large number of simultaneously collected samples at several time points before, during, and after RSV infection, offered several obstacles in recruitment and retention of subjects. We believe the relatively intensive commitment required on the part of young parents, often with the stress of caring for their first infant, resulted in a higher than anticipated attrition rate among the birth cohort. To adjust for loss of subjects in the birth cohort, we modified the enrollment strategy to include infants seen in the outpatient setting for acute respiratory illness or during routine visits for immunization when they were noted to have very mild respiratory signs. This modification of the enrollment strategy resulted in enrollment of mildly ill RSV-infected infants, thus, allowing development of a GRSS with a scale of 0 to 10 that spans the entire severity spectrum. This tool will allow us to utilize the severity endpoint as either a dichotomous or continuous variable in our analyses of the various parameters measured. As these datasets are completed, they will be made available in public repositories for use by other investigators.

### Strengths

We chose to assay host gene expression responses as a primary indicator of health status of subjects. The methods we have developed to interrogate nasal gene expression, which appears to be a reasonable surrogate of the airway and is capable of being recovered from infants of varying age and health status, is highly novel. This approach is clearly capable of providing significant new opportunities to define the molecular status of the airway in ill infants, a major impediment to prior research of infant lung diseases [80]. We also chose to separate peripheral blood cells into major subtypes before interrogation of their gene expression. Although this raised some significant technical challenges, they have been overcome [37,50]. Furthermore, this approach allows us to better achieve our primary objectives of defining the biology of the system and how it is affected by disease state.

### Future Plans

Ultimately, we plan to integrate each of the datasets using disease severity (GRSS) as the clinical outcome, allowing us to identify and account for interactions among the various data types during the infant’s response to RSV infection. These analyses should provide important insights into to the complexity of RSV disease pathogenesis and potentially to novel interventions to alter RSV severity.

## Abbreviations

ANOVA: analysis of variance
AsPIRES: Assessing Predictors of Infant RSV Effects and Severity
CD: cluster of diffirentiation
DMSO: deoxymethysulfoxide
GRSS: Global Respiratory Severity Score
IgG: immunoglobulin G
NK: natural killer
OTU: Operational taxonomic unit
PBMC: peripheral blood mononuclear cell
PCA: principle component analysis
PCR: polymerase chain reaction
PICU: pediatric intensive care unit
qPCR: quantitative polymerase chain reaction
RGH: Rochester General Hospital
RNA: ribonucleic acid
RNAseq: ribonucleic acid sequencing
RSV: respiratory syncytial virus
RT-PCR: reverse-transcriptase polymerase chain reaction
SaO2: room air oxygen saturation
Th2: type 2 helper
URMC: University of Rochester Medical Center
